# A Single Centre Analysis of Clinical Characteristics and Treatment of Endocrine Pancreatic Tumours

**DOI:** 10.1155/2015/538948

**Published:** 2015-06-08

**Authors:** M. T. Adil, R. Nagaraja, V. Varma, N. Mehta, V. Kumaran, S. Nundy

**Affiliations:** Department of Surgical Gastroenterology and Liver Transplantation, Sir Ganga Ram Hospital, New Delhi 110060, India

## Abstract

*Background*. Endocrine Pancreatic Tumours (PENs) are rare and can be nonfunctioning or functioning. They carry a good prognosis overall though high grade lesions show a relatively shorter survival. The aim of the current study is to describe a single centre analysis of the clinical characteristics and surgical treatment of PENs.* Patients and Methods*. This is a cohort analysis of 40 patients of PENs who underwent surgery at Sir Ganga Ram Hospital, New Delhi, India, from 1995 to 2013. Patient particulars, clinical features, surgical interventions, postoperative outcome, and followup were done and reviewed. The study group was divided based on grade (G1, G2, and G3) and functionality (nonfunctioning versus functioning) for comparison.* Results*. PENs comprised 6.3% of all pancreatic neoplasms (40 of 634). Twenty-eight patients (70%) had nonfunctioning tumours. Eighteen PENs (45%) were carcinomas (G3), all of which were nonfunctioning. 14 (78%) of these were located in the pancreatic head and uncinate process (*P* = 0.09). The high grade (G3) lesions were significantly larger in size than the lower grade (G1 + G2) tumours (7.0 ± 3.5 cms versus 3.1 ± 1.6 cms, *P* = 0.007). Pancreatoduodenectomy was performed in 18 (45%), distal pancreatectomy in 10 (25%), and local resection in 8 (20%) and nonresective procedures were performed in 4 patients (10%). Fourteen patients (35%) had postoperative complications. All G3 grade tumours which were resected had positive lymph nodes (100%) and 10 had angioinvasion (71%). Eight neoplasms (20%) were cystic, all being grade G3 carcinomas, while the rest were solid. The overall disease related mortality attributable to PEN was 14.3% (4 of 28) and for malignant PENs was 33.3% (4 of 12) after a mean follow-up period of 49.6 months (range: 2–137 months).* Conclusion*. Majority of PENs are nonfunctioning. They are more likely malignant if they are nonfunctioning and large in size, show cystic appearance, and are situated in the pancreatic head. Early surgery leads to good long term survival with acceptable postoperative morbidity.

## 1. Introduction

Pancreatic endocrine neoplasms (PENs) arise from pluripotent cells of the pancreas [1, 2] and constitute less than 2% of all pancreatic tumours [3–8]. They commonly occur in the fourth and fifth decades of life and carry a slight female preponderance [9].

PENs can be functioning and present with symptoms attributable to oversecretion of offending hormones or nonfunctioning where no such paraneoplastic symptoms occur [2, 10]. Nonfunctioning tumours usually present with abdominal pain or features of advanced disease such as jaundice and a palpable abdominal mass [11, 12]. Some patients may be diagnosed asymptomatically after an incidental detection following abdominal imaging done for some other reason [4, 13].

Diagnosis of functioning PENs is confirmed by detection of elevated levels of offending hormones in the serum and serum chromogranin A levels [14, 15]. Helical computed tomography is the current noninvasive imaging modality of choice for the initial evaluation of PENs [16–18]. Endoscopic ultrasound may allow for imaging of small lesions not detected by CT but is invasive and unnecessary in most cases [4]. Intraoperative ultrasound (IOUS) helps in peroperative localization of small functioning PENs and is more sensitive than other noninvasive methods [18, 19]. Somatostatin receptor scintillography (SRS) and Positron emission tomography (PET) scan can be used to detect the doubtful lesions as well as extrahepatic spread [20].

The mainstay of treatment for PENs is surgery [2, 10]. Local resection is considered for small PENs. Larger lesions and those suspicious of malignancy are treated with formal pancreatic resections while surgical debulking is reserved for large tumours with locoregional spread and selected patients with metastasis. The overall prognosis of PENs is good; however survival rates are low in high grade PENs [21].

The aim of the current study is to describe a single centre experience with surgical management of PENs including analysis of demographics, presenting characteristics, operative course, surgical morbidity, and followup.

## 2. Methods

### 2.1. Data Collection

We did a cohort analysis from a prospectively maintained database of patients who underwent pancreatic surgery for neoplasms of the pancreas at Sir Ganga Ram Hospital, New Delhi, India, from 1995 to 2013, and using pathological reports and preoperative CT scan as gold standard, we identified 40 patients with PENs.

A review of medical files, electronic records, operative notes, and discharge summaries was done to collect information regarding demographics, clinical features, and laboratory results. Patients were categorized into functioning and nonfunctioning PENs based on their clinical behavior, pathology reports, and serum radioimmunoassay for offending hormones. Preoperative CT scans were used to characterize PENs and record neoplasm size and location.

Type of surgery performed, operative findings, postoperative hospital stay, and complications were recorded. The complications noted were delayed gastric emptying (intolerance to oral feeds requiring nasogastric aspiration after the sixth postoperative day), postoperative fever (temperature more than 100°F sustained enough to require medication and search for its cause), wound infection (purulent discharge from the main wound with positive culture report), and intra-abdominal abscess (purulent fluid of any amount inside the abdomen requiring percutaneous drainage or laparotomy). A pancreatic fistula was defined as drainage of fluid with amylase levels at least 3 times the normal serum amylase levels from the third postoperative day. Postoperative mortality was defined as death within the same hospital admission or within 30 days of surgery. Followup was done at 3 and 6 months and then at 6 monthly intervals and evidence of disease recurrence or disease related mortality was recorded.

Evaluation of the pathology reports was done to note the tumour size, nodal status, and evidence of distant spread and staging of the disease was done as per the WHO (2010)/TNM/AJCC staging classification for pancreatic exocrine and endocrine tumours [22]. Microscopic findings were used to categorize the malignant potential of every specimen based on the WHO 2010 grading classification of neuroendocrine neoplasms of the digestive system [22] and the alternative Ki-67 proliferation index cut-off value of 5% and 20% was used to grade the specimens [21] (Table 1). Specimen analysis was done for presence of tumour location, microscopic vascular invasion, and presence of cystic component and positive resection margins and staining characteristics were noted.

### 2.2. Statistical Analysis

Results were interpreted as mean (SD) and range where applicable. Comparison between groups was made in percentages and numbers or using independent *t*-test or chi-square test depending on the variables and distribution of data. These tests were 2-sided and a *P* value less than 0.05 was considered statistically significant.

## 3. Results

634 patients underwent surgery for pancreatic neoplasms between 1995 and 2013 at our institute of which we identified 40 patients (6.3%) with pancreatic endocrine neoplasms (PENs). The mean (SD) age of the patients was 48.6 (15.52) years (range: 32–79 years). The disease was almost equal in incidence between the sexes with 22 (55%) males.

Twenty-eight (70%) tumours were nonfunctioning and twelve (30%) were functioning of which 10 were insulinomas and 2 were gastrinomas. All our tumours were sporadic and there was no association with MEN 1 or any other syndromes.

The location of PENs is enumerated in Table 2.

Eight (20%) PENs were tumours of grade G1, fourteen (35%) were of G2, and eighteen (45%) were carcinomas of grade G3 as per the WHO (2010) classification [22]. When the alternative Ki-67 index cut-off value of 5% and 20% was applied, the number of grade G1 and G2 tumours was 10 and 12, respectively [21].

All eighteen carcinomas were nonfunctioning. Fourteen (78%) carcinomas were located in the pancreatic head and uncinate process and two each were in the tail and neck + body combined.

The mean tumour size was 5.0 cms (range: 0.5–12.0 cms). Comparison of mean tumour sizes with respect to functionality and grade is enumerated in Table 3.

Abdominal pain was the most common presenting feature of nonfunctioning PENs occurring in 22 of 28 patients (78%). Four (10%) cases were diagnosed incidentally with no symptoms attributable to PENs. The presenting features are enumerated in Table 4.

The mean serum haemoglobin level was 11.6 g/dL (range: 6.6–15.9 g/dL) and the mean serum bilirubin level was 0.8 mg/dL (range: 0.4−2.0 mg/dL).

The types of surgeries performed are presented in Table 5 and operative data in Table 6.

Eight (20%) patients had local invasion at the time of surgery of which 4 were unresectable (10%). All of them were carcinomas (grade G3). Two tumours arising from the pancreatic head had infiltration of the transverse colon and the duodenum and were treated with a right colectomy along with pancreatoduodenectomy. Two tumours arising from the tail had locally invaded into the stomach and a total gastrectomy was done along with distal pancreatic resection.

All fourteen malignant tumours which were resected had positive lymph nodes (100%) and 10 had microscopic angioinvasion (71%). 8 neoplasms (20%) showed cystic appearance on gross pathology. One patient following pancreatoduodenectomy showed a positive resection margin (R1 resection).

**Figure 1:**
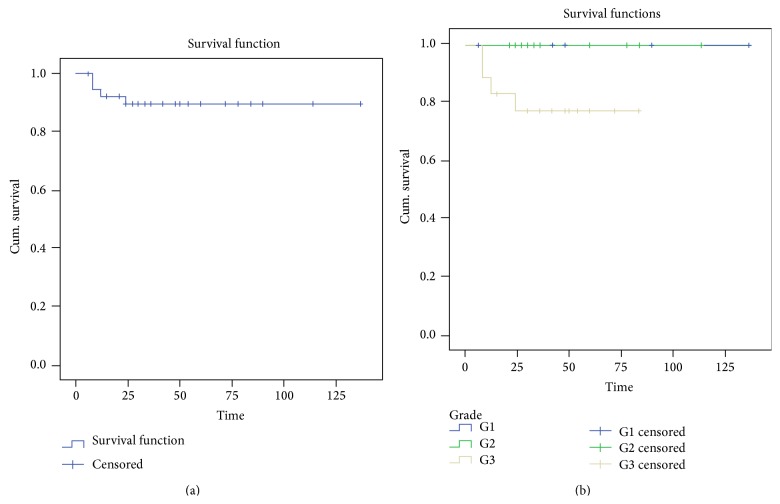
Kaplan-Meier survival curve for pancreatic endocrine neoplasms (*n* = 40) overall and by grade. (a) The Kaplan-Meier survival curve is shown with the number of patients at risk. (b) The neoplasms were grouped by the grade of the tumour.

The mean duration of postoperative hospital stay was 18 days (range: 6–60 days). Postoperative complications occurred in 14 patients (35%) after surgery, all of whom underwent pancreatoduodenectomy. Eight (20%) patients had delayed gastric emptying, four (10%) patients had postoperative wound infection, two of whom had undergone a concomitant right colectomy, two (5%) patients developed postoperative fever, and two (5%) had an intra-abdominal abscess which was drained percutaneously. Six (15%) patients developed pancreatic fistula, all of whom responded to conservative treatment. No complication occurred in patients who underwent distal pancreatectomy or local resection. No perioperative deaths occurred in our study. The overall disease specific mortality attributable to PEN was 14.3% (4 of 28). Disease related mortality for nonfunctioning PEN was 25% (4 of 16) and for malignant PENs was 33.3% (4 of 12) (Figure 1).

## 4. Discussion

The first description of pancreatic endocrine neoplasms was an islet cell adenoma by Nichols more than 100 years back [23]. PENs have been historically diagnosed based on functionality and nonfunctioning lesions were rarely reported. Functioning tumours were reported to comprise between 50 and 85% of PENs in the past [12, 24]. However recent data shows nonfunctioning PENs to comprise between 70 and 90% [2, 17]. PENs have also commonly been known to affect the body and tail of the pancreas [17, 25]. Our findings show that 70% of PENs are nonfunctioning and these were significantly more common in the right pancreas than its functioning counterpart with 22 tumours (79%) arising from the head and uncinate process (*P* = 0.006). Interestingly we also noted that 75% (30 of 40) of PENs were operated at our institute in the second half (2004−2013) of the study period. This temporal shift in incidence was seen only in nonfunctioning tumours and we believe that the increased detection is due to improved imaging and better understanding of the disease.

Multidetector, triple phase, and thin slice computed tomography detects nonfunctioning PENs with reported sensitivities between 80 and 95% [2, 26, 27]. Functioning neoplasms are typically diagnosed at smaller sizes due to hormone oversecretion [4], while nonfunctioning tumours are detected when they are large enough to cause pain or mass effect [12, 28]. We found that the mean duration of symptoms of functioning PENs (12 days (range: 7–21 days)) was significantly shorter than its nonfunctioning counterpart (35 days (range: 14–90 days)) (*P* = 0.002) and attribute this to the acute hormonal crisis in functioning PENs. Because functioning tumours can present at smaller sizes, conventional CT imaging may fail to localize them. Somatostatin receptor scintillography and more recently PET scan have shown excellent sensitivity in localizing PENs [29–32]. For appropriate staging and evaluation of extra pancreatic disease for therapeutic decision making, somatostatin receptor scintillography (SRS) with ^111^In-octreotide has a sensitivity and specificity of 90% and 80%, respectively [33–35]. PET scan with ^68^Ga-labelled somatostatin analogue (DOTA^0^-Phe^1^-Tyr^3^)-octreotide (DOTATOC) is superior to conventional ^18^F-fluorodeoxyglucose PET as well as ^111^In-DTPA-octreotide (^111^In-DTPAOC) SPECT in imaging PENs [20, 36]. Endoscopic ultrasound (EUS) can detect small lesions (<2 cms) not detected by CT scan and can predict lymph node involvement with a sensitivity of more than 90% [37–40]. Combining EUS with fine needle aspiration (FNA) can give cellular diagnosis by revealing neuroendocrine cells [17]. However, we rarely do FNA preoperatively in patients planned for surgery as negative or inconclusive results would not affect our decision to operate. We however routinely do intraoperative ultrasound (IOUS) to detect small functional lesions.

Prediction of malignancy in PENs is a subject of active research and many classification systems and guidelines have been suggested over the years. The old WHO (2004) classification divided PENs into tumours (benign or uncertain behavior) and carcinomas (well differentiated or poorly differentiated) [41]. Carcinomas were defined by presence of local invasion or metastasis [41]. The recent WHO (2010) classification, however, defines a carcinoma by the presence of G3 grade and not the presence of local invasion and metastasis as in their previous classification [22]. To add further prognostic significance, few studies have suggested that the predictive cut-off value of Ki-67 proliferation index at 5% and 20% instead of the WHO (2010) and ENETS suggested cutoff at 2% and 20% probes better into prognosis and survival differences between the grades, especially between grades G1 and G2 [21, 42, 43]. When we applied the alternative Ki-67 index (MIB-1 antibody) cutoff in our study, the number of G1 and G2 grade tumours changed to 10 and 12, respectively; however no change in the outcome could be interpreted in our study by applying the alternative cutoff, as no adverse event or deaths were recorded in either of the groups. According to a study, of the 37 malignant PENs operated in the cohort, 21 (57%) had positive lymph nodes and 20 (54%) had histological angioinvasion [17]. The literature once said that histological angioinvasion in a PEN is sufficient to diagnose it as a malignancy [44]. However, while, according to the old WHO (2004) classification, PENs with microscopic evidence of angioinvasion are a well-differentiated endocrine tumour (WDET) of uncertain behaviour and not necessarily a carcinoma and lymph nodal spread is criteria of carcinoma [41], the recent WHO (2010) staging classification places lymph nodal involvement in Stage IIb and above but makes no mention of the role of angioinvasion in the classification of PENs [22]. In our study, all 14 operated carcinomas had at least one positive lymph node (mean = 3, range 1−20) and ten (71%) had evidence of microscopic angioinvasion. When we compared this difference, we found that lymph nodal involvement was not a significantly more common finding than angioinvasion in pancreatic endocrine carcinomas (100% versus 71%) (*P* = 0.15). It would be interesting to study the role of microscopic angioinvasion in the prediction of malignancy and survival on a larger cohort in the future.

The role of peripancreatic nodal clearance in PENs also remains an area of controversy. Vagefi et al. suggest enucleation or segmental resections for benign tumours less than 3 cms and state that the role of peripancreatic lymphadenectomy is not clear given the biological nature of these tumours [25]. However, the ENETS guidelines suggest a followup strategy after a staging lymphadenectomy for nonfunctioning tumours less than 2 cms [45]. In our study, of the fourteen resected pancreatic endocrine carcinomas, 8 had tumour sizes of 3 cms or less. Six of them underwent formal pancreatic resections while two patients with small nonfunctioning lesion in the body of the pancreas underwent local resection with peripancreatic nodal clearance which on histology showed G3 grade with positive lymph nodes (well-differentiated endocrine carcinoma according to the old WHO (2004) classification). Our strategy is to do peripancreatic nodal clearance even in small nonfunctioning tumours of suspicious nature since we believe that lymph node involvement can occur even in small malignant neoplasms of high grade which may mistakenly appear innocent. However, whether nodal clearance actually translates into survival benefit in these groups of patients needs to be studied on a larger population.

Kazanjian et al. found that the overall complication rate was 48% after pancreatoduodenectomy, 12.5% after distal pancreatectomy, and none after local resection [17]. We observed an overall complication rate of 35%, all occurring after pancreatoduodenectomy and none after distal pancreatectomy and local resection (*P* < 0.0001).

Cystic PENs have been linked to carcinomas of larger sizes [46, 47]. We found 8 patients in our study who had a cystic component on gross pathology, all of which were nonfunctioning carcinomas of G3 grade. The mean (SD) size of cystic PENs was 9.0 (2.0) cms (range: 8−12 cms) which was significantly larger than that of solid tumours (3.9 (2.7) cms (range: 0.5−6 cms)) (*P* = 0.0006). It would be interesting to study the prognosis of PENs based on cystic changes on a larger population since it appears to be exclusively present in high grade lesions.

Pancreatic endocrine neoplasms are associated with good 5-year survival rates ranging between 77% and 89% in different studies [17, 25]. The most significant factors influencing survival are the grade, stage of disease, and completeness of resection [12, 21, 26, 48, 49]. In our series, one patient had a positive resection margin (R1 resection). Of the 28 (70%) patients whose follow-up data was available, four (14%) deaths were reported. All were grade G3 solid tumours. Twenty-four of 28 (85.7%) are alive after a mean follow-up period of 49.6 months (range: 2–137 months).

## 5. Conclusions

- PENs should be kept in the differential diagnosis of all pancreatic space occupying lesions.
- Nonfunctioning tumours are more common than functioning lesions and they are more commonly diagnosed in the pancreatic head.
- Pancreatic endocrine carcinomas (grade G3) should be suspected if the lesion is large in size and nonfunctioning, occurs in the pancreatic head, and has cystic changes.
- High grade (G3) and solid lesions appear to carry a worse prognosis.
- Early surgery leads to good long term survival with acceptable postoperative morbidity.

## Strengths of the Study

- Published data on Endocrine Pancreatic Tumours is rare and this study provides an insight into the disease.
- This study provides a comprehensive single institution analysis of data of a rare disease collected over 18 years.
- This study provides a future paradigm for analysis over larger data and multicenter research.

## Weaknesses of the Study

- The study is a cohort analysis of a rare disease with a relatively small sample size.
- 30% of patients were lost to followup.

## Figures and Tables

**Table 1:** WHO grading classification (2010) of pancreatic neuroendocrine tumours [22].

Low grade neuroendocrine tumour—G1	<2 mitoses/10 HPF and/or ≤2% Ki-67 proliferation index

Intermediate grade neuroendocrine tumour—G2	2–20 mitoses/10 HPF and/or 3%–20% Ki-67 proliferation index

High grade neuroendocrine carcinoma—G3	>20 mitoses/10 HPF and/or >20% Ki-67 proliferation index

Alternative Ki-67 cut-off value, 5% and 20%, between G1/G2 and G2/G3, respectively.

**Table 2:** Location of 40 pancreatic endocrine neoplasms.

Tumour location	Nonfunctioning	Functioning	Total (%)
	*n* = 28	*n* = 12	*n* = 40
Head	18	4	22 (55)
Head + uncinate	2	—	2 (5)
Body	—	4	4 (10)
Tail	4	4	8 (20)
Uncinate	2	—	2 (5)
Neck + body	2	—	2 (5)

**Table 3:** Comparison of tumour sizes with respect to functionality and grade.

Tumour characteristics	Mean (SD)	*P* value
	[in cm]	
Nonfunctioning (*n* = 28)	5.6 (3.4)	**0.06**
Functioning (*n* = 12)	3.1 (1.9)	

G1 + G2 (*n* = 22)	3.1 (1.6)	**0.007**
G3 (*n* = 18)	7.0 (3.5)	

**Table 4:** Presenting complaints of pancreatic endocrine neoplasms.

Presenting feature	Nonfunctioning (*n* = 28)	Functioning (*n* = 12)	Percentage (%) of total (*n* = 40)
Abdominal pain	22	0	55

Abdominal mass	4	0	10

Vomiting	2	2	10

Hepatomegaly	2	0	5

Gastric outlet obstruction	2	0	5

Hypertension	2	0	5

Neuroglycopenic symptoms	0	10	25

Dyspepsia	4	2	15

**Table 5:** Surgeries performed for pancreatic endocrine neoplasms.

Type of surgery	Number (%) of nonfunctioning PENs (*n* = 28)	Number (%) of functioning PENs (*n* = 12)
(1) Resective procedures		
(1.1) Local resection	4 (14.2)	4 (33.3)
(1.2) Pancreatoduodenectomy	16 (57.1)	2 (17.7)
	[2 SMV resection; 6 MR]	
(1.3) Distal pancreatectomy	4 (14.2)	6 (50%)
		[2, spleen preservation]

(2) Nonresective procedures		
(2.1) Gastrojejunostomy	2 (7.1)	—
(2.2) Open biopsy	2 (7.1)	—

SMV = superior mesenteric vein; MR = Machado reconstruction.

**Table 6:** Comparisons of the resective procedures with respect to demographics, tumour size, postoperative complications, and postoperative hospital stay.

Characteristics	PD (*n* = 18)	DP (*n* = 10)	LR (*n* = 8)	*P* value
Age [mean (SD)] (years)	46.7 (13.6)	47.6 (25.3)	54.5 (9.9)	0.29

Sex				
Males	6	8	4	
Females	12	2	4	

Tumour size [mean (SD)] (cms)	5.0 (3.6)	4.0 (2.9)	3.6 (1.8)	0.39

Postoperative complications (%)	77	0	0	<0.0001

Postoperative hospital stay (days)	19.7 (5.6)	18.8 (4.8)	11.3 (3.3)	0.0015

PD = pancreatoduodenectomy; DP = distal pancreatectomy; LR = local resection.
